# Stimulation of vagus nerve for patients with disorders of consciousness: a systematic review

**DOI:** 10.3389/fnins.2023.1257378

**Published:** 2023-09-15

**Authors:** Xiaoyang Dong, Yunliang Tang, Yifan Zhou, Zhen Feng

**Affiliations:** Department of Rehabilitation Medicine, The First Affiliated Hospital of Nanchang University, Nanchang, Jiangxi, China

**Keywords:** disorders of consciousness, vagus nerve stimulaiton, transcutaneous auricular vagus nerve stimulation, vagus nerve magnetic modulation, vegetative state/unresponsive wakefulness state, minimally conscious state, systematic review

## Abstract

**Purpose:**

The purpose of this study is to evaluate the efficacy and safety of stimulating the vagus nerve in patients with disorders of consciousness (DOCs).

**Methods:**

A comprehensive systematic review was conducted, encompassing the search of databases such as PubMed, CENTRAL, EMBASE and PEDro from their inception until July 2023. Additionally, manual searches and exploration of grey literature were performed. The literature review was conducted independently by two reviewers for search strategy, selection of studies, data extraction, and judgment of evidence quality according to the American Academy of Cerebral Palsy and Developmental Medicine (AACPDM) Study Quality Scale.

**Results:**

A total of 1,269 articles were retrieved, and 10 studies met the inclusion criteria. Among these, there were three case reports, five case series, and only two randomized controlled trials (RCTs). Preliminary studies have suggested that stimulation of vagus nerve can enhance the levels of DOCs in both vegetative state/unresponsive wakefulness state (VS/UWS) and minimally conscious state (MCS). However, due to a lack of high-quality RCTs research and evidence-based medical evidence, no definitive conclusion can be drawn regarding the intervention’s effectiveness on consciousness level. Additionally, there were no significant adverse effects observed following stimulation of vagus nerve.

**Conclusion:**

A definitive conclusion cannot be drawn from this systematic review as there was a limited number of eligible studies and low-quality evidence. The findings of this systematic review can serve as a roadmap for future research on the use of stimulation of vagus nerve to facilitate recovery from DOCs.

## Introduction

Disorders of consciousness (DOCs) refer to prolonged periods of impaired awareness following severe brain injuries or neurological impairments, such as traumatic brain injury (TBI), stroke, hypoxic–ischemic encephalopathy (HIE) and other related conditions (Dostovic et al., 2012; Eapen et al., 2017; Malone et al., 2019). The DOCs can be classified into four categories based on their neurobehavioral function: coma, vegetative state/unresponsive wakefulness state (*VS*/UWS), minimally conscious state (MCS), and the emergence from MCS to higher consciousness level, namely eMCS (Cortese et al., 2023; Li et al., 2023). Comas are states of unconsciousness characterized by a lack of arousal and consciousness. In comas, spontaneous or stimulus-induced arousal is absent, and there is no opening of the eyes, as well as sleep–wake cycles are lost during EEG testing (Ardeshna, 2016). The term *VS*/UWS denotes the condition characterized by the preservation of fundamental brainstem reflexes and the sleep–wake cycle, accompanied by either spontaneous or induced eye opening, albeit without conscious awareness (Monti et al., 2010). The MCS referring to a severely altered state of consciousness in which there is minimal but definite evidence of awareness of self or surroundings, characterized by inconsistent but clearly discernible behavioral evidence of consciousness and can be distinguished from coma and *VS*/UWS by documenting the presence of specific behavioral features not found in either of these conditions (Giacino et al., 2002). MCS includes MCS+ and MCS–, MCS+ syndrome should be marked by reproducible evidence of any one of the following behaviors: command-following, intelligible verbalization, or intentional communication, while MCS– included automatic motor behaviors, object manipulation, localizing objects in space, localizing noxious stimuli, visual pursuit, and visual fixation, but no evidence of receptive or expressive language function (Thibaut et al., 2020).

There are currently alternative treatment options for DOCs, including pharmacological treatments such as amantadine, sensory stimulation, hyperbaric oxygen therapy, and neuromodulation (Septien and Rubin, 2018; Thibaut et al., 2019). Neuromodulation, encompassing non-invasive brain stimulation techniques like transcranial direct current stimulation (tDCS) and repetitive transcranial magnetic stimulation (rTMS), as well as invasive brain stimulation methods like deep brain stimulation (DBS) and spinal cord stimulation (SCS), holds significant potential as a therapeutic avenue for various neurological disorders, including drug-resistant epilepsy, depression, and DOCs (Perez-Carbonell et al., 2020; Marwaha et al., 2023).

In recent years, the utilization of stimulation of vagus nerve techniques, such as invasive vagus nerve stimulation (VNS), transcutaneous auricular vagus nerve stimulation (taVNS), and vagus nerve magnetic modulation (VNMM), has garnered significant interest among neuroscientists for the treatment of consciousness disorders. These techniques present a promising neuromodulatory therapeutic approach for the recovery of patients with DOCs. Nevertheless, a comprehensive systematic review evaluating the effectiveness and safety of stimulation of vagus nerve in the context of DOCs is currently lacking.

Hence, considering the significance of this matter and the dearth of empirical evidence substantiating the efficacy of any rehabilitative intervention for individuals with DOCs, the primary objective of this study was to investigate the effectiveness of stimulation of vagus nerve in treating DOCs. Furthermore, we aimed to ascertain any potential untoward consequences associated with this therapeutic approach.

## Methods

The present systematic review was carried out following the guidelines specified in the Preferred Reporting Items for Systematic Review and Meta-Analysis (PRISMA) statement (Moher et al., 2010).

### Participants

Individuals of diverse age, gender, and ethnicity, who have been diagnosed with coma, unresponsive wakefulness syndrome/vegetative state, minimally conscious state, extended minimally conscious state, and/or exhibit impaired consciousness as determined by assessment tools such as the Glasgow Coma Scale (GCS) or the Coma Recovery Scale-Revised (CRS-R), are included in this study. The scope of this study encompasses patients diagnosed with DOCs, focusing on clinical research and excluding animal-based experimental investigations.

### Intervention

The stimulation of the vagus nerve, whether through implanted VNS or non-invasive taVNS, as well as other methods of vagus nerve stimulation such as VNMM by rTMS, is not dependent on the specific parameters employed (such as type of current, frequencies, amplitudes, and intensity) or the duration of treatment.

### Outcome

The study primarily examined the impact of electrostimulation treatment on the level of consciousness, as measured by appropriate scales such as CRS-R and GCS. Secondary outcomes focused on potential adverse events, including changes in heart rate, blood pressure, respiratory rate, and/or saturation. Additionally, brain assessment techniques such as functional magnetic resonance imaging (fMRI), somatosensory evoked potentials (SEP), brainstem auditory evoked potentials (BAEP), and cerebral blood flow (CBF) were utilized.

### Type of studies

We have exclusively incorporated clinical studies, for example, randomized controlled clinical trials (RCTs), case reports, case series, and other relevant sources. It should be emphasized that animal studies are not included.

### Information sources

Adhering to the latest guidelines for updating systematic reviews, we have specifically opted for articles published after May 1, 2008, to ensure the provision of novel evidence based on necessity and priority. Our objective was to encompass studies from international English-language journals until July 10, 2023, pertaining to stimulation of vagus nerve in DOCs. The primary sources were acquired through comprehensive exploration of biomedical databases, gray literature, and meticulous examination of bibliographies of all deemed pertinent articles.

The biomedical databases examined in this study encompassed CENTRAL (Cochrane Central Register of Controlled Trials), MEDLINE (accessible through PubMed), EMBASE, and PEDro. Additionally, we conducted searches in databases containing clinical trial protocols, sought out unpublished or ongoing trials, and performed citation link searches using research bibliographies obtained from the aforementioned biomedical databases.

### Search strategy

**Figure 1:**
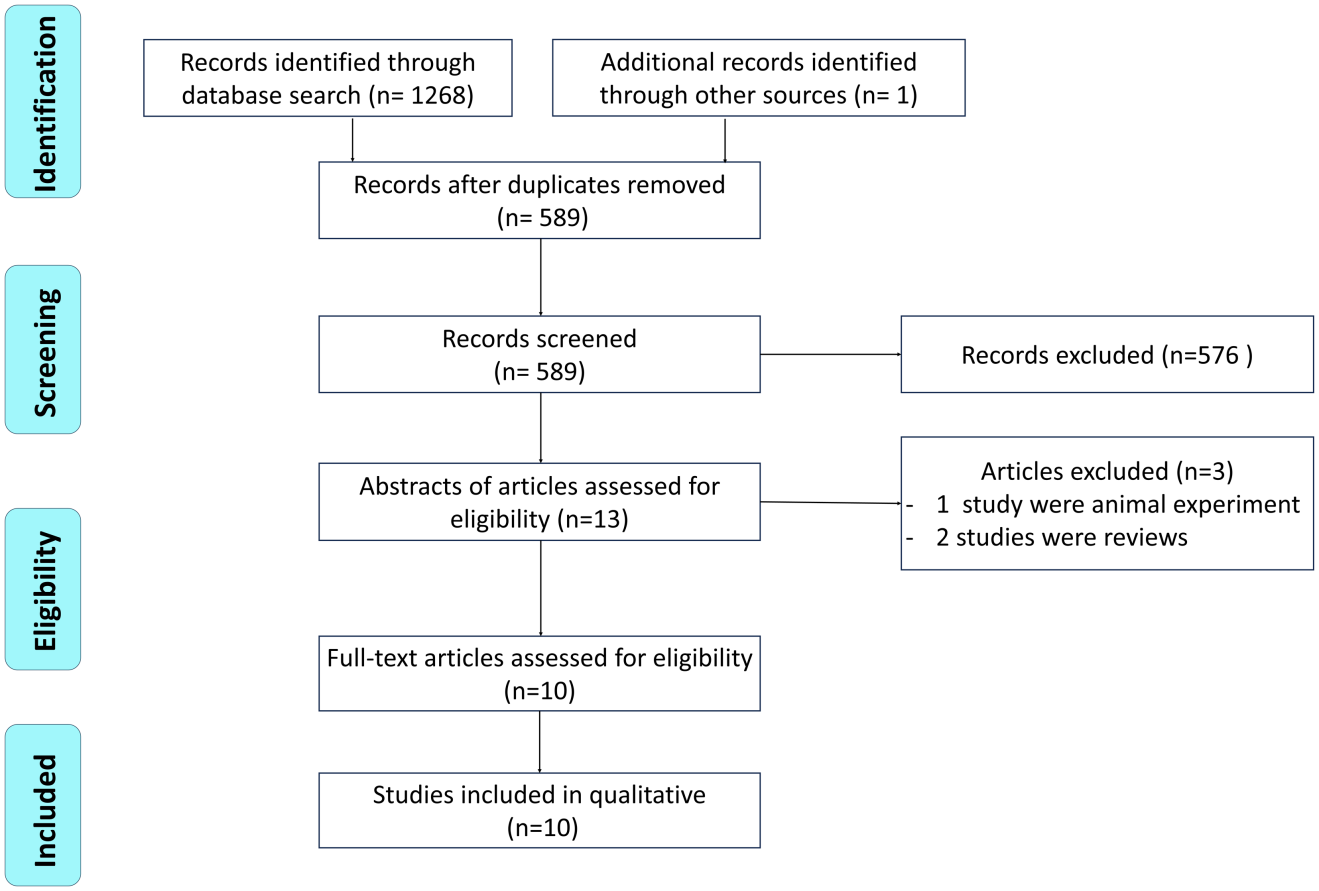
PRISMA flowchart.

The search on Pubmed was: (“Consciousness Disorders” [Mesh] OR “Consciousness” [Mesh] OR Conscious* OR Unresponsive* OR Unconsciousness OR Coma* OR Unawareness OR Vegetative) AND (“Vagus Nerve” [Mesh] OR “Vagus nerve”). The search on CENTRAL was: (MeSH descriptor: [Consciousness] OR MeSH descriptor: [Consciousness Disorders] OR Coma* OR Conscious* OR Unresponsive* OR Unconsciousness OR Unawareness OR Vegetative) AND (MeSH descriptor: [Vagus Nerve] OR “Vagus nerve”). The search strategy on Embase was: (Conscious OR Unresponsive OR Unconsciousness OR Coma OR Unawareness OR Vegetative) AND “Vagus nerve.” For PEDRo we used only the term “Vagus.” An example of a PRISMA flow sheet is included, showing how the search strategy is put in place (Figure 1).

### Study selection

The articles were selected by two authors (CV and FT) through a sequential analysis of the title, abstract, and full text, if accessible. Any conflicts arising between the two authors were resolved through comparison or, if necessary, the involvement of a third author (DF).

### Data collection process

The data from the individual studies were obtained using a paper-based template created by two authors (CV and FD). Any discrepancies in the collected data were resolved through comparison or with the involvement of a third author (TI). Additionally, the variables extracted from each article included the participants’ characteristics, intervention details, outcome measures along with their respective follow-up periods, and the obtained results.

## Results

### General aspects

A bibliographic research process was conducted, resulting in the identification of 1,268 studies. An additional study was found through citation chaining strategies. After removing duplicates, a total of 680, the title and abstract of 589 articles were screened. From this screening, 576 records were excluded, leaving 13 articles for further evaluation through reading the full text. Three papers were subsequently excluded, as one study involved animal experimentation and two studies were reviews. Ultimately, a total of 10 studies were selected. The study selection process is presented in the PRISMA flowchart depicted in Figure 1. The characteristics of each individual study have been extracted and summarized in Table 1.

**Table 1:** Characteristics of included studies.

Study	Type	Patients no.	Etiology	Stimulation devices	Stimulation side and site	Stimulation parameter	Data cycle	Clinical results	Side effect	Brain evaluation
Yu et al. (2017)	Case report	1	HIE	taVNS	Bilateral cymba	20 Hz, <1 ms, 4–6 mA	30 min, twice daily, 50 days	*VS* → MCS CRS-R: 6 → 13	–	fMRI: DMN connectivity↑
Corazzol et al. (2017)	Case report	1	TBI	VNS, Cyberonics Inc	Left surgical implantation of vagus nerve	30 Hz, 500 us, 0.25–1.5 mA	30s on/5 min off, 6 months	*VS* → MCS CRS-R: 5 → 10	–	EEG: theta band power ↑, wSMI: ↑, PET: activity in occipito-parieto-frontal and basal ganglia regions ↑
Hakon et al. (2020)	Case series	5	DAI after TBI	taVNS, Nemos®	Left cymba conchae	25 Hz, 250 us, 30s on/30s off, 0.5–1 mA	4 h, once a day, 8 weeks	2 MCS → EMCS 1 of 3 *VS* → MCS	Intermittent itching of the ear (1 patient)	–
Noe et al. (2020)	Case series	14	TBI: 7, HIE: 4, Hemorrhage: 3	taVNS, Parasym® CE	Left tragus	20 Hz, 250 us, 1.5 mA	30 min, twice daily, 5 days a week, 4 weeks	CRS-R: 5 of 8 MCS patients↑, 6 *VS*/UWS patients no changed	None	–
Xiang et al. (2020)	Case series	10	TBI: 4, HIE: 1, Hemorrhage: 5	VNS, G112, PINS Medical	Left surgical implantation of vagus nerve	20–30 Hz, 250–500 us, 0.1–3.5 mA	30s on/5 min off, 6 months	CRS-R: 9 of 10 patients↑	None	–
Yu et al. (2021)	Case series	10	HIE: 5, Hemorrhage: 3, TBI: 2	taVNS	Cymba conchae	20 Hz, 0.5 ms, 4–6 mA	30 min, twice daily, 4 weeks	CRS-R: 6 of 7 *VS* patients↑, 2 of 3 MCS patients↑	–	fMRI: CBF ↑ in auditory responded group
Osinska et al. (2022)	Case report	1	TBI	taVNS, Nemos®	Cymba conchae	25 Hz, 0.25 ms, 25 V, 30s on /30s off, 0.2–1.5 mA	4 h, once daily, 6 months	CRS-R: 4 → 13	–	EEG: alpha range ↑
Wang et al. (2022)	Case series	17	Hemorrhage: 9, HIE: 3, TBI: 5	VNMM, TMS (magneuro 60 stimulator)	Left mastoid	10 Hz	20 min, once daily, 5 days per week, 4 weeks	CRS-R: 7.88 ± 2.93 → 11.53 ± 4.94 GCS: 7.65 ± 1.90 → 9.18 ± 2.65	None	SEP: 1 patient improved from grade II to grade I. BAEP: grade I: 3 → 5, grade II: 8 → 9, grade III: 4 → 1, grade IV: 2 → 2
Yifei et al. (2022)	RCT	12	Stroke: 8, TBI: 2, Anoxic: 2	taVNS, Huatuo brand electronic acupuncture	Bilateral auricular concha	20 Hz, <1 ms, 4–6 mA	30 min, twice daily, 4 weeks	CRS-R: no significant improvement	–	EEG: delta band↑
Zhou et al. (2023)	RCT	57	Stroke: 30, TBI: 27	taVNS, Changzhou Rishena Medical Device	Left auricular concha	20 Hz, 200 us, intensity, 15	30 min, twice daily, 6 days per week, 4 weeks	CRS-R: significant improvement for MCS patients	None	–

DAI, diffuse axonal injury; TBI, traumatic brain injury; HIE, hypoxic ischemic encephalopathy; VNS, vagus nerve stimulation; taVNS, transauricular vagus nerve stimulation; VNMM, vagus nerve magnetic modulation; CRS-R, revised coma recovery scale; GCS, glasgow scale; minimally conscious state; VS/UWS, vegetative state/unresponsive wakefulness syndrome; EEG, electroencephalogram; DMN, default mode network; fMRI, functional magnetic resonance imaging; SEP, somatosensory evoked potentials; BAEP, brainstem auditory evoked potentials; CBF, cerebral blood flow.

### Study design and quality

All 10 articles consisted of prospective studies that examined the effects of vagus nerve stimulation on patients with DOCs, encompassing both *VS*/UWS and MCS. The evaluation of the articles’ quality was conducted using the American Academy of Cerebral Palsy and Developmental Medicine (AACPDM) Study Quality Scale (Petrus et al., 2008) (refer to Table 2), the Clinical Relevance Tool for Case Studies, and the Quality, Rigor or Evaluative Criteria tool.

**Table 2:** American Academy of Cerebral Palsy and Developmental Medicine (AACPDM) levels of evidence.

Levels of evidence	Study design
I	Systematic review of randomized controlled trials (RCT) Large RCT (with narrow confidence interval)
II	Smaller RCTs (with wider confidence intervals) Systematic reviews of cohort studies “Outcomes research” (very large ecologic studies)
III	Cohort studies (must have concurrent control group) Systematic reviews of case–control studies
IV	Case series Cohort study without concurrent control group (e.g., with historical control group) Case–control study
V	Expert opinion Case study or report Bench research Expert opinion based on theory or physiologic research Common sense/anecdotes

Two out of the 10 studies included in this analysis were randomized-controlled trials, which were categorized as level II evidence according to the AACPDM level of evidence scale (Yifei et al., 2022; Zhou et al., 2023). Five articles consisted of case series that lacked an active control group or sham group, resulting in their classification as level IV evidence (Hakon et al., 2020; Noe et al., 2020; Xiang et al., 2020; Yu et al., 2021; Wang et al., 2022). The remaining three articles were case reports that exhibited limited individual study quality, thus classified as level V evidence (Corazzol et al., 2017; Yu et al., 2017; Osinska et al., 2022).

### Study samples

A total of 128 patients diagnosed with DOCs, encompassing both female and male individuals, were included in the various studies conducted. These studies focused on patients classified as either in a *VS*/UWS or in a MCS. One study exclusively examined MCS subjects (Xiang et al., 2020), while four case series reports (Hakon et al., 2020; Noe et al., 2020; Xiang et al., 2020; Wang et al., 2022) and two randomized controlled trials (Yifei et al., 2022; Zhou et al., 2023) included both *VS*/ UWS and MCS patients. Furthermore, the etiology of DOCs encompassed conditions such as HIE, TBI, hemorrhage, and stroke.

### Stimulation of vagus nerve protocols

The primary methods of stimulating the vagus nerve encompass invasive VNS, non-invasive taVNS, and VNMM. Among the articles reviewed, two employed VNS (Corazzol et al., 2017; Xiang et al., 2020), seven utilized taVNS (Yu et al., 2017; Hakon et al., 2020; Noe et al., 2020; Yu et al., 2021; Osinska et al., 2022; Yifei et al., 2022), and one employed VNMM through rTMS (Wang et al., 2022). The stimulation parameters for VNS included a sinusoidal waveform, pulse width ranging from 250 to 500 μs, a frequency of 20–30 Hz, and an amplitude ranging from 0.1 to 3.5 mA, targeting the left vagus nerve. For taVNS, the parameters consisted of a pulse width of 200–500 μs, a frequency of 20 to 25 Hz, an amplitude ranging from 0.1 to 6 mA, and targeting either the left or bilateral cymba conchae. In the case of VNMM, a frequency of 10 Hz was applied through rTMS to the left mastoid. In most studies, stimulation protocol lasted for 4 weeks, once or twice a day, for 30 min.

### Consciousness assessment

The evaluation of consciousness disorders in these papers primarily encompasses behavioral assessments, such as the CRS-R and the GCS, as well as brain functional evaluations, including EEG, evoked potentials, fMRI, and positron emission tomography (PET). All of the studies employed the CRS-R as the primary outcome measure, with only one study utilizing the GCS as a secondary outcome measure (Wang et al., 2022). These studies reported significant improvements in CRS-R scores following intervention, except for Yifei’s study, which did not demonstrate any significant improvement (Yifei et al., 2022). Several studies have reported alterations in the connectivity of the default mode network (DMN) (Yu et al., 2017) and CBF in patients, as observed through fMRI examinations (Yu et al., 2021). Furthermore, EEG (Corazzol et al., 2017; Osinska et al., 2022), evoked potentials (Wang et al., 2022) and PET (Corazzol et al., 2017) have also provided evidence of brain changes following stimulation.

### Adverse effects

Out of the total of 10 studies examined, only one study conducted by Hakon et al. (2020) systematically addressed the adverse effects. This particular study reported that a single patient experienced intermittent itching of the ear during stimulation, although the severity of this symptom did not significantly impact the level of stimulation.

## Discussion

The primary objective of this systematic review was to assess the efficacy of stimulation of vagus nerve in facilitating the recovery of consciousness among patients diagnosed with DOCs. Additionally, the secondary objective was to evaluate any potential adverse effects associated with this therapeutic intervention.

A total of 10 articles were gathered, comprising three case reports, five case series, and two RCTs (Table 1). In 2017, Yu et al. conducted a study in which they documented the case of a 73-year-old female patient who experienced respiratory and cardiac arrests (Yu et al., 2017). The patient exhibited partial recovery of impaired consciousness, transitioning from a *VS*/UWS to a MCS after undergoing taVNS for a duration of 4 weeks. The patient’s level of consciousness improved from 6 points (*VS*/UWS) to 13 points (MCS) on the CRS-R following the 4-week taVNS intervention. Additionally, fMRI revealed an increase in the functional connectivity of the DMN after the taVNS treatment. In the same year, Corazzol et al. conducted an invasive VNS procedure on a patient with *VS*/UWS caused by lesions in multiple regions of the brain (Corazzol et al., 2017). This patient had been in a *VS*/UWS state for over 15 years. VNS was administered to the patient’s left vagus nerve for a duration of 6 months following the onset of treatment. The application of VNS resulted in a significant increase in the patient’s CRS-R scores, rising from 5 to 10 points. Furthermore, the patient’s condition transitioned from *VS*/UWS to MCS.

In 2020, Hakon et al. conducted a study to examine the feasibility and safety of transcutaneous taVNS in patients with DOCs following TBI (Hakon et al., 2020). The study included three patients in a *VS*/UWS and two patients in a MCS who had experienced diffuse axonal injury more than 28 days prior. Following the 8-week taVNS intervention, three patients demonstrated improvement in the CRS-R, with two MCS patients transitioning to a higher level of consciousness and one *VS*/UWS patient progressing to MCS. Another study conducted by Noe et al. (2020) to examine the feasibility, safety and therapeutic effects of taVNS treatment in 14 patients (six with *VS*/UWS and eight with MCS) who had been diagnosed with DOCs for more than 6 months following brain injury (seven patients with TBI, four patients with anoxia, and three patients with hemorrhage). Throughout the 4 weeks leading up to taVNS treatment, there were no observed alterations in the CRS-R scores of the patients. However, at the conclusion of the one-month follow-up, there was a significant increase in the CRS-R scores. It is noteworthy that none of the patients diagnosed with *VS*/UWS exhibited any modifications in their CRS-R scores, whereas five out of the eight patients diagnosed with MCS displayed a progressive rise in their CRS-R scores over the course of this study. Xiang et al. (2020) conducted a study to examine the therapeutic effects of VNS on patients with MCS. The study included 10 MCS patients who had experienced TBI in four cases, hemorrhage in five cases, and HIE in one case. These patients were evaluated more than 5 months after their initial injury and had undergone VNS implantation on the left vagus nerve. Following 3 months of VNS, a notable disparity was detected in the overall CRS-R scores when compared to the initial measurements. Subsequently, after 6 months of VNS intervention, CRS-R evaluations consistently exhibited substantial enhancements, leading to the emergence of one patient from the MCS.

In 2021, Yu et al. conducted a preliminary study to examine the cerebral hemodynamic correlates of taVNS in the restoration of consciousness (Yu et al., 2021). The study included 10 patients with DOCs resulting from severe brain damage, specifically anoxia (five patients), hemorrhage (three patients), and traumatic brain injury (two patients). The patients who exhibited a response to auditory stimuli demonstrated a favorable outcome on the GCS following the four-week taVNS treatment. Conversely, the patients who did not respond to auditory stimuli experienced unfavorable outcomes. Simultaneously, taVNS increased CBF of multiple brain regions in the DOCs patients who responded to auditory stimuli.

In 2022, Osinska et al. documented a case study involving a patient who exhibited a restoration of impaired consciousness following 6 months of taVNS treatment (Osinska et al., 2022). The subject, a 28-year-old female, had been diagnosed with *VS*/UWS based on a four-point assessment on the CRS-R subsequent to a TBI that had occurred 6 years earlier. Notably, the patient’s CRS-R score significantly improved from 4 to 13 points after approximately 100 days of taVNS therapy, suggesting a transition from *VS*/UWS to MCS or potentially even MCS+. Wang et al. conducted an evaluation on the impact of VNMM on a group of 17 patients diagnosed with DOCs (Wang et al., 2022). The patients were categorized as follows: 4 patients with *VS*/UWS, 11 patients with MCS, and 2 patients in a coma state. The underlying cause of the DOCs in these patients was acquired brain injury, with three patients experiencing HIE, nine patients with hemorrhage, and five patients with TBI. The results of both the CRS-R and the GCS demonstrated notable enhancements in patients with DOCs following 4 weeks treatment with VNMM. Additionally, improvements were observed in somatosensory evoked potentials and brainstem auditory evoked potentials. Yifei et al. (2022) investigated the effect of taVNS in 12 patients with DOCs (*VS*/UWS, seven patients and MCS, five patients) due to acquired brain injury (stroke, eight patients; anoxia, two patients and TBI, two patients). TaVNS was applied for 14 days and none of the patients exhibited notable advancements on the CRS-R scale; nevertheless, the resting state EEG power spectrum indicated a decline in the energy of the delta band and an elevation in the energy of the beta band among patients diagnosed with MCS, as opposed to those diagnosed with *VS*/UWS.

In 2023, Zhou et al. conduct a randomized controlled clinical trial to investigated the therapeutic efficacy and safety of taVNS in patients with DOCs (Zhou et al., 2023). The study included a total of 57 patients with DOCs, comprising 25 patients in a *VS*/UWS and 32 patients in a MCS, all of whom had acquired brain injuries, specifically 30 patients with stroke and 27 patients with TBI. The findings from this initial study offer preliminary evidence suggesting that taVNS could potentially serve as a safe and effective method for facilitating the restoration of consciousness in patients diagnosed with MCS, but not in those with *VS*/UWS.

In general, the utilization of stimulation of vagus nerve in individuals with DOCs demonstrated effectiveness, as evidenced by positive outcomes observed in 9 of 10 studies (Corazzol et al., 2017; Yu et al., 2017; Hakon et al., 2020; Noe et al., 2020; Xiang et al., 2020; Yu et al., 2021; Osinska et al., 2022; Wang et al., 2022; Zhou et al., 2023). Additionally, only one study reporting an itching sensation in the ear (Hakon et al., 2020). Moreover, seven studies investigating alterations in brain activity subsequent to stimulation of vagus nerve reported favorable results, employing various techniques such as fMRI, EEG, PET, and SEP. In terms of the application methods, the application site and time schedules of taVNS were found to be consistent across seven studies, with the cymba conchae being the chosen site, sessions lasting 30 min, and occurring twice daily. However, there was considerable variation in the treatment period, ranging from 4 weeks to 6 months. In relation to the electrical stimulation parameters, the frequency remained consistent across all studies at 20–25 Hz. However, there was variability in both the pulse width (ranging from 200 to 1,000 μs) and intensity (ranging from 0.1 to 6 mA). Furthermore, two studies employed an invasive method of VNS through left surgical implantation (Corazzol et al., 2017; Xiang et al., 2020), while one study utilized TMS on the left mastoid (Wang et al., 2022). Nevertheless, these publications are unable to yield a definitive conclusion due to the insufficiency of high-quality evidence. Primarily, the reporting quality of these studies is generally inadequate, as none of the included studies have provided a confidence interval or a measure of variance. This limitation has hindered the possibility of conducting a meta-analysis. Additionally, only two studies are RCTs, both with small sample sizes, encompassing 12 patients (Yifei et al., 2022) and 57 patients (Zhou et al., 2023), respectively. Hence, it is plausible to assert that the aforementioned studies may have lacked sufficient statistical power, thereby accounting for the limited occurrence of statistically significant outcomes. Additionally, it is worth noting that the longest duration of follow-up in these studies was merely 4 weeks post-incident, which presents a noteworthy constraint. This limitation becomes particularly significant when considering that a definitive diagnosis of *VS*/UWS necessitates a minimum period of 12 months following a non-traumatic event and 6 months following a traumatic event (Laureys et al., 2004; Roquilly et al., 2021).

However, the existing literature does not provide any evidence of level one support for a rehabilitation treatment aimed at enhancing consciousness recovery in patients with DOCs (Kondziella et al., 2020; Edlow et al., 2021). Consequently, it is justifiable to argue that, in light of the absence of reported adverse effects in the study conducted by Hakon et al. (2020) and the theoretical framework proposed in animal studies, stimulation of vagus nerve holds promise as a potential treatment for patients with DOCs, as supported by our own clinical experience with these individuals. Currently, there is an increasing number of reported study protocols for non-invasive taVNS in the context of DOCs (Cheng et al., 2023; Zhai et al., 2023). These protocols aim to design randomized controlled trials with large multicenter samples to assess the efficacy and safety of taVNS therapy for DOCs, as well as investigate the neural anatomy associated with taVNS during the process of consciousness recovery.

There have been a multitude of scholarly reports discussing the potential mechanisms through which the stimulation of the vagus nerve may augment wakefulness. Previous research conducted by our team has shown that VNS facilitates the restoration of consciousness in rats experiencing coma following TBI. Additionally, it has been observed that the upregulation of neurotransmitters, specifically orexin-A, in the prefrontal cortex may contribute to the wake-promoting effects of VNS (Dong et al., 2018; Dong and Feng, 2018). Meanwhile, it is plausible that VNS could mitigate brain damage following traumatic brain injury through the suppression of inflammation, oxidative stress, and apoptosis (Tang et al., 2020; Wang et al., 2021). Moreover, the spinoreticular segment of the vagus nerve pathway establishes connections with neurons of the ascending reticular activating system (ARAS), a pivotal structure responsible for sustaining wakefulness (Yuan and Silberstein, 2016). This observation implies the potential for VNS to exert an impact on the ARAS through vagus nerve stimulation. Furthermore, augmentation of CBF (Kunii et al., 2021), activation of neurotrophic factors, and modulation of synaptic plasticity (Follesa et al., 2007; Biggio et al., 2009) may also contribute to these effects.

The data presented in this systematic review hold significant importance in informing future research regarding the utilization of vagus nerve stimulation in patients with DOCs. Notably, a gap in the existing literature has been identified, necessitating the need for well-designed RCTs to address this gap. It is crucial to emphasize that forthcoming RCTs should strictly adhere to rigorous methodological standards, particularly in terms of selecting appropriate allocation concealment techniques and effectively managing missing data. Additionally, it is imperative that these trials adhere to the established reporting guidelines as outlined by Moher et al. (2012). It is also essential to incorporate the calculation of confidence intervals and the measurement of statistical variability to enhance the feasibility of future meta-analyses. In addition, it is recommended to employ alternative evaluation methods, such as evoked potentials, encephalogram, and functional near-infrared spectroscopy (fNIRS), in addition to the current use of the CRS-R in RCTs. Finally, subsequent studies should rigorously examine the potential negative consequences associated with vagus nerve stimulation, including but not limited to bradycardia, laryngismus, dyspepsia, dyspnea, heightened coughing, pain, voice modulation, paresthesia, headache, pharyngitis, infection, and others (Ben-Menachem, 2001; Wheless et al., 2018).

However, this systematic review is subject to certain limitations. The inclusion of studies with limited availability of information and poor methodological quality hinders the ability to establish conclusive findings. Additionally, we encountered challenges in obtaining the unpublished protocol of a substantial RCTs from a clinical trial protocol database. Despite attempts to contact the author for information regarding the study’s publication date and access to raw data, no response was received.

Based on the findings of this systematic review, it is not feasible to establish conclusive recommendations regarding the application of vagus nerve stimulation as a treatment for patients with DOCs. This limitation arises primarily from the scarcity of studies available in the existing literature and their inadequate methodological rigor. Consequently, further research is necessary before definitive conclusions can be reached regarding the efficacy of VNS or taVNS in the management of DOCs. Further research is imperative, encompassing the key attributes of rigorous methodology, appropriate sample size selection, utilization of outcome measures with enhanced content validity for assessing consciousness levels, comprehensive investigation into potential adverse effects, and long-term monitoring of follow-up outcomes, while considering the prognosis of consciousness disorders.

## Author contributions

XD: Conceptualization, Funding acquisition, Writing – original draft, Writing – review & editing, Formal analysis. YT: Methodology, Software, Investigation, Validation, Project administration, Resources, Writing – review & editing. YZ: Data curation, Investigation, Software, Validation, Writing – review & editing. ZF: Conceptualization, Resources, Supervision, Validation, Visualization, Data curation, Funding acquisition, Writing – review & editing.

## Funding

The author(s) declare financial support was received for the research, authorship, and/or publication of this article. This study received financial support from the National Natural Science Foundation of China (82260457 and 82202811), the Jiangxi Provincial Natural Science Foundation (20224BAB216042) and the Science and Technology Department of Jiangxi Province Project (20212BAG70023).

## Conflict of interest

The authors declare that the research was conducted in the absence of any commercial or financial relationships that could be construed as a potential conflict of interest.

## Publisher’s note

All claims expressed in this article are solely those of the authors and do not necessarily represent those of their affiliated organizations, or those of the publisher, the editors and the reviewers. Any product that may be evaluated in this article, or claim that may be made by its manufacturer, is not guaranteed or endorsed by the publisher.
